# Comparison of painful temporomandibular disorders, psychological characteristics, sleep quality, and oral health-related quality of life of patients seeking care before and during the Covid-19 pandemic

**DOI:** 10.1186/s12903-023-03158-w

**Published:** 2023-07-01

**Authors:** Adrian Ujin Yap, Jie Lei, Chengge Liu, Kai-Yuan Fu

**Affiliations:** 1 Center for TMD & Orofacial Pain, Peking University School & Hospital of Stomatology, No. 22 Zhong Guan Cun South Ave, Beijing, 100081 China; 2 Department of Dentistry, Ng Teng Fong General Hospital, and Faculty of Dentistry, National University Health System, Singapore, Singapore, Singapore; 3 National Dental Research Institute Singapore, National Dental Centre Singapore and Duke-NUS Medical School, Singapore Health Services, Singapore, Singapore; 4 Department of Oral & Maxillofacial Radiology, Peking University School & Hospital of Stomatology, No. 22 Zhong Guan Cun South Ave, Beijing, 100081 China; 5 National Center for Stomatology and National Clinical Research Center for Oral Diseases, Beijing, China; 6 National Engineering Research Center of Oral Biomaterials and Digital Medical Devices, Beijing, China; 7 Beijing Key Laboratory of Digital Stomatology, Beijing, China

**Keywords:** Temporomandibular disorders, Covid-19, Cohort effect, Psychological distress, Sleep quality, Quality of life

## Abstract

**Background:**

Literature concerning Temporomandibular disorders (TMDs) and the Covid-19 pandemic is limited and disparate findings related to TMD frequencies, psychological distress, and quality of life were presented. This study investigated the prevalence of painful Temporomandibular disorders (TMDs) and compared the psychological, sleep, and oral health-related quality of life profiles of patients seeking TMD care before and during the Covid-19 pandemic.

**Methods:**

Data were accrued from consecutive adult patients 12 months before (BC; control) and during (DC; case group) the Covid-19 pandemic. The Diagnostic Criteria for TMDs (DC/TMD), Depression, Anxiety, Stress Scales (DASS)-21, Pittsburgh Sleep Quality Index (PSQI), and Oral Health Impact Profile (OHIP)-TMDs were utilized and statistical analysis was performed using Chi-square/non-parametric tests (α = 0.05).

**Results:**

The prevalence of painful TMDs was 50.8% before and 46.3% during the pandemic. Significant differences in PSQI and OHIP component scores were discerned between the BC and DC groups contingent on TMD pain. Total-DASS was moderately correlated to total-PSQI/OHIP (*r*_*s*_ = 0.41–0.63).

**Conclusion:**

The covid-19 pandemic did not appear to exacerbate psychological distress but affected sleep and increased unease over TMD dysfunction.

## Background

Temporomandibular disorders (TMDs) refer to a cluster of medical/dental problems affecting the stomatognathic system. The cardinal features of TMDs include jaw joint/muscle pain, headaches, jaw joint sounds, and jaw opening or closing difficulties [1, 2]. As stipulated by the evidence-based Diagnostic Criteria for TMDs (DC/TMD), common TMDs can be classified into pain-related and intra-articular conditions which are painful and non-painful correspondingly [3]. Pain-related conditions consist of temporomandibular joint (TMJ) arthralgia, masticatory muscle myalgia, headache attributed to TMDs, whereas intra-articular conditions comprise TMJ disc displacements, degenerative joint disease, and subluxation [3]. Though the prevalence of TMDs was reported to range from 5 to 16% in the general population, up to 75% of people have TMD signs/symptoms [4, 5]. Women, notably those of reproductive age, have a greater risk of TMDs and constitute the bulk of TMD patients [4, 6, 7]. TMDs, especially when painful, are associated with poor sleep and impaired oral health-related quality of life (OHRQoL) [8–11]. The complex etiology of TMDs follows the “biopsychosocial model of illness” and contributing factors encompass gene-environment interactions, sex hormones, poor general health, macro/micro-trauma including oral parafunction, somatization, and psychological distress [12–17]. Recent systematic reviews/meta-analyses have indicated a high occurrence of psychological distress in patients seeking TMD care with depression and anxiety being more frequently distributed among those with painful TMDs [16, 17].

“Black swan” events, like the Coronavirus Disease 2019 (Covid-19) pandemic, refer to rare and unforeseen phenomena with significant impact on society as a whole. Covid-19 pneumonia was first detected in late December 2019 and was declared a global pandemic in early Mar 2020 by the World Health Organization due to its rapid spread all over the world [18]. Until the introduction of Covid vaccines in December 2020, the measures adopted by most countries involved stringent partial-to-total lockdown, social distancing, and active disease surveillance (test-trace-isolate [TTI]). Such mitigating strategies to contain Covid-19 outbreaks were employed in China which had a “zero-Covid” policy up to Dec 2022 [19]. Though the citywide TTI approach is highly effective in lowering Covid infections and deaths, it disrupts daily life and has far-reaching existential, social, economic, and health consequences, including elevated levels of psychological problems [20–22]. The latter could trigger or aggravate TMD signs/symptoms ensuing in treatment-seeking [23].

Literature concerning TMDs and the Covid-19 pandemic is still limited. The few cross-sectional studies conducted during and one year after the Covid-19 pandemic indicated greater frequencies of symptoms and higher levels of psychological distress among individuals with TMDs [24–27]. Furthermore, both sleep quality and OHRQoL were also found to be diminished and related to TMDs [28, 29]. Nevertheless, the two available prospective studies involving TMD patients yielded varied findings with one demonstrating greater psychological distress and another specifying no worsening of pain intensity and OHRQoL during the Covid-19 pandemic [30, 31]. Besides socio-cultural and other local differences, the disparate observations could also be explained by variances in TMD subtypes [30–33].

Based on the above premises, the objectives of this study were three folds: (i) to examine the prevalence of painful TMDs before and during the Covid-19 pandemic, (ii) to compare the psychological characteristics, sleep quality, and OHRQoL of patients seeking TMD care during the two periods, and (iii) to establish the influence of the Covid-19 pandemic as an “impact event” and other variables on TMD expression. The research hypotheses were: (a) the proportion of patients with painful TMDs increased during the Covid-19 pandemic, (b) patients seeking care during the Covid-19 pandemic had higher levels of psychological distress, sleep disturbance, and poorer OHRQoL, and (c) the Covid-19 pandemic, amid other variables, increased the odds of painful TMDs.

## Methods

### Study design

This project is part of ongoing research investigating the physical and psychosocial effects of TMDs, supported by the Biomedical Institutional Review Board of the Peking University School of Stomatology (project number: PKUSSIRB-201,732,009).

Data from consecutive adult patients seeking care at a university-based TMD/orofacial pain clinic 12 months before (Mar 2019 to Feb 2020) and during (Mar 2020 to Feb 2021) the Covid-19 pandemic were accrued. Patients who sought care before the pandemic served as the control group, whereas those who pursued care during the pandemic were the case group. Effectively, two patient cohorts were evaluated according to their time of presentation and assessment. At least 169 participants were required for the control as well as case groups. This was derived based on a 95% confidence level, 5% precision, 48% prevalence of painful TMDs, and 300 new cases anticipated annually [33]. The inclusion criteria were age ≥ 18 years old, proficiency in the Chinese language, and the presence of TMD symptoms, specifically TMJ/masticatory muscle pain, headaches, TMJ noises, closed, and/or open locking. The exclusion criteria were a history of orofacial trauma/orthognathic surgery and craniofacial deformities, the presence of systemic joint diseases, non-musculoskeletal causes of orofacial pain, debilitating psychological, or cognitive disorders, drug/substance abuse, and illiteracy. Involvement in the study was voluntary and all eligible participants provided informed consent. At the intake visit, demographic/medical information was collected and a survey comprising the Chinese versions of the DC/TMD Symptom Questionnaire (SQ), Depression, Anxiety, Stress Scales (DASS)-21, Pittsburgh Sleep Quality Index (PSQI), and Oral Health Impact Profile (OHIP)-TMDs was administered [34–40].

### TMD subtypes/categories

After completing the questionnaires, participants underwent an intake examination which was conducted according to the DC/TMD protocol by a formally trained and calibrated TMD specialist [34]. Palpation/movement pain, pain location/referral, jaw deviation/movement, and TMJ noises were assessed and orthopantomography and/or cone-beam computed tomography were used to verify intra-articular conditions. Magnetic resonance imaging was performed only on a “need basis” due to its high cost and indications include the persistence of symptoms after conservation treatment, functional jaw alterations, and the suspicion of neoplastic processes. DC/TMD axis I physical diagnoses were rendered using the DC/TMD algorithms/diagnostic tree based on symptom history from the SQ, clinical, and radiographic findings. The three possible TMD diagnostic groupings based on TMD conditions were: pain-related (PT) – positive for arthralgia, myalgia, and/or headache; intra-articular (IT) – positive for TMJ disc displacements, degenerative joint disease, and/or subluxation; and combined (CT) disorders – positive for PT plus IT. Depending on the presence or absence of TMD pain, the control and case groups were dichotomized into those with painful (PT and CT) and non-painful (solely IT) TMDs.

### Study measures

Psychological distress was appraised with the 21-item DASS-21 which contained three subscales, specifically depression, anxiety, and stress. The reliability, validity, and bifactor structure (comprising a general factor for distress [total-DASS] and the three emotional constructs) are well established [35, 36, 41]. Seven items were allotted to each of the three subscales and scored on a four-point response scale ranging from “did not apply to me at all” = 0 points to “applied to me very much or most of the time” = 3 points. While total-DASS scores spanned from 0 to 63 points, subscale scores varied from 0 to 21 points with greater scores indicating higher levels of general distress, depression, anxiety, and stress. Cut-off points for categorizing the severity (normal to extremely severe) of the three subscales are described in the DASS manual [35].

Sleep quality was examined with the 19-item PSQI which assessed seven aspects of sleep, namely subjective sleep quality, sleep latency, sleep duration, sleep efficiency, sleep disturbances, use of sleep medication, and daytime dysfunction. The PSQI has good psychometric properties and is widely utilized in TMD and other research [42, 43]. Items are mostly scored on a four-point response scale extending from “not during the past month/very good” = 0 points to “three or more times a week/very bad” = 3 points. Components scores were computed following defined rules and added to derive total-PSQI scores which ranged from 0 to 21 points. Greater total-PSQI scores indicated worse sleep quality and a score of ≥ 6 served as the cut-point for poor sleep [38].

OHRQoL was appraised with the 22-item OHIP-TMDs which contained seven domains, specifically functional limitation, physical pain, psychological discomfort, physical disability, psychological disability, social disability, and handicap [39, 40]. Its measurement properties were confirmed in clinical as well as non-clinical populations with TMDs [39, 40, 44, 45]. Two to five items were allotted to each of the seven domains and scored on a five-point response scale extending from “never” = 0 points to “very often” = 4 points. Total-OHIP scores ranged from 0 to 88 points whilst domain scores varied from 0 to 20 depending on the number of items involved. Greater total and domain OHIP scores indicated worse/poorer OHRQoL.

### Statistical analyses

Statistical assessments were carried out with the SPSS Statistics software version 27.0 (IBM Corporation, Armonk, New York, USA) with the significance level set at 0.05. Qualitative data were described as frequencies with percentages and evaluated with the Chi-square test. Quantitative data were reported as means/medians with standard deviations (SDs)/interquartile ranges (IQRs) and examined for normality with the Shapiro-Wilk’s test. As non-normal distributions were noted, the Mann-Whitney U test and Spearman’s rank order correlations were applied. Correlation coefficients (*r*_*s*_) of 0.1, 0.4, and 0.7 served as cut-off points for weak, moderate, and strong relationships between variables [46]. Univariate and multivariate logistic regression analyses were conducted to establish the predictors of painful and non-painful TMDs including the Covid-19 pandemic. A stepwise variable selection procedure was used in the multivariate modeling with a threshold of p < 0.10 for excluding insignificant ones. Results were depicted as odds ratios (ORs) with 95% confidence intervals (95% CIs).

## Results

Of the 632 patients seen between Mar 2019 to Feb 2021, 116 did not meet the eligibility criteria due to age (< 18 years old). Although none of the eligible patients declined study participation, 75 returned incomplete surveys ensuing in an effective response rate of 85.5%. Table 1 presents the demographic characteristics of the total sample, control, and case groups. The mean age of the final study sample (n = 441) was 33.2 ± 13.7 years with no significant differences in age between patients who sought treatment before (BC; control group) and during (DC; case group) the Covid-19 pandemic. Women comprised 81.2% of the participants and gender distributions between the BC and DC groups were statistically insignificant. The prevalence of painful TMDs was 50.8% (13.1% PT/37.7% CT conditions) before and 46.3% (17.1% PT/29.2% CT conditions) during the pandemic. No significant differences in the frequencies of PT, CT, and IT conditions as well as painful and non-painful TMDs were discerned between the BC and DC groups.

**Table 1:** Demographic characteristics of the control (before covid) and case (during Covid) groups

Variables	All patients n (%)	Before Covid (BC)	During Covid (DC)	P-value
Number of TMD patients				
n (%)	441 (100)	236 (100)	205 (100)	
**Age**				
*Mean (SD)*	33.24 (13.73)	33.44 (13.95)	33.01 (13.49)	0.662^
*Median (IQR)*	29.00 (15.00)	29.00 (13.75)	28.00 (16.00)	-
**Gender**				
*Women, n (%)*	358 (81.2)	190 (80.5)	168 (82.0)	0.699*
*Men, n (%)*	83 (18.8)	46 (19.5)	37 (18.0)	
*Female;male* *(F:M) ratio*	4.3	4.1	4.6	-
**TMD conditions**				
*Pain-related, n (%)*	66 (15.0)	31 (13.1)	35 (17.1)	0.143*
*Combined pain-related plus intra-articular, n (%)*	149 (33.8)	89 (37.7)	60 (29.3)	
*Intra-articular, n (%)*	226 (51.2)	116 (49.2)	110 (53.7)	
**TMD categories**				
*Painful TMDs, n (%)*	215 (48.8)	120 (50.8)	95 (46.3)	0.345*
*Non-painful TMDs, n (%)*	226 (51.2)	116 (49.2)	110 (53.7)	

SD = standard deviation; IQR = interquartile range. Results of ^Mann-Whitney U and *Chi-square tests. Bold indicates p < 0.05

Table 2 shows the mean/median DASS-21 and PSQI scores for the BC and DC groups with painful and non-painful TMDs. For both TMD categories, no significant differences in total and subscale DASS scores were observed between the BC and DC groups. However, when TMD pain was present, the DC group reported significantly greater sleep latency and use of sleep medication scores than the BC group (DC > BC). Conversely, in the presence of non-painful TMDs, significantly greater sleep duration and efficiency scores were observed in the BC group (BC > DC). Patients with painful TMDs (PF) had significantly greater stress scores than their counterparts with non-painful TMDs (NP) both before and during the pandemic (PF > NP). Significant differences in total-DASS and depression scores were noted only before the pandemic (PF > NP). Subjective sleep quality and daytime dysfunction scores did not vary much between PF and NP patients both before and during the pandemic. While sleep disturbance scores differed substantially before the pandemic (PF > NP), significant disparities in total-PSQI, sleep latency, sleep duration, sleep efficiency, and use of sleep medication scores between PF and NP patients were detected during the pandemic (PF > NP).

**Table 2:** Mean/median DASS/PSQI scores for the control and case groups with painful and non-painful TMDs.

		Painful TMDs			Non-painful TMDs		
Variables		Before Covid (BC)	During Covid (DC)	P-value^	Before Covid (BC)	During Covid (DC)	P-value^
**DASS-21**							
***Total-DASS***	Mean (SD)	31.03 (28.90)	27.85 (25.75)	0.583	22.69 (24.24)	21.51 (20.24)	0.847
	Median (IQR)	21.00 (38.00) ^a^	22.00 (34.00) ^A^		12.00 (32.00) ^b^	18.00 (26.00) ^A^	
***Depression***	Mean (SD)	9.02 (10.43)	7.37 (8.70)	0.522	5.67 (8.03)	5.18 (6.29)	0.534
	Median (IQR)	6.00 (14.00) ^a^	4.00 (12.00) ^A^		2.00 (10.00) ^b^	4.00 (8.00) ^A^	
***Anxiety***	Mean (SD)	10.00 (9.17)	8.55 (8.60)	0.209	7.90 (7.98)	7.93 (7.83)	0.854
	Median (IQR)	8.00 (13.00) ^a^	6.00 (10.00) ^A^		6.00 (9.50) ^a^	6.00 (8.00) ^A^	
***Stress***	Mean (SD)	12.02 (11.08)	11.94 (10.50)	0.928	9.12 (9.91)	8.40 (8.52)	0.981
	Median (IQR)	10.00 (18.00) ^a^	10.00 (14.00) ^A^		6.00 (13.50) ^b^	6.00 (10.00) ^B^	
**PSQI**							
***Total-PSQI***	Mean (SD)	6.93 (4.04)	7.26 (4.12)	0.567	6.34 (3.43)	5.84 (2.93)	0.404
	Median (IQR)	6.00 (5.00) ^a^	7.00 (5.00) ^A^		5.50 (4.75) ^a^	6.00 (3.00) ^B^	
***Subjective sleep quality***	Mean (SD)	1.16 (0.78)	1.19 (0.88)	0.853	1.22 (0.72)	1.11 (0.64)	0.335
	Median (IQR)	1.00 (1.00) ^a^	1.00 (1.00) ^A^		1.00 (1.00) ^a^	1.00 (0) ^A^	
***Sleep latency***	Mean (SD)	1.08 (1.04)	1.35 (0.97)	**0.039** DC > BC	1.13 (0.93)	1.07 (0.98)	0.565
	Median (IQR)	1.00 (2.00) ^a^	1.00 (1.00) ^A^		1.00 (2.00) ^a^	1.00 (2.00) ^B^	
***Sleep duration***	Mean (SD)	1.18 (0.85)	1.05 (0.93)	0.235	1.06 (0.88)	0.76 (0.70)	**0.011** BC > DC
	Median (IQR)	1.00 (2.00) ^a^	1.00 (2.00) ^A^		1.00 (2.00) ^a^	1.00 (1.00) ^B^	
***Sleep efficiency***	Mean (SD)	0.53 (0.97)	0.62 (0.99)	0.370	0.47 (0.84)	0.22 (0.48)	**0.029** BC > DC
	Median (IQR)	0 (1.00) ^a^	0 (1.00) ^A^		0 (1.00) ^a^	0 (0) ^B^	
***Sleep disturbances***	Mean (SD)	1.13 (0.52)	1.06 (0.54)	0.310	0.98 (0.46)	0.97 (0.50)	0.766
	Median (IQR)	1.00 (0) ^a^	1.00 (0) ^A^		1.00 (0) ^b^	1.00 (0) ^A^	
***Use of sleep medication***	Mean (SD)	0.31 (0.86)	0.59 (1.09)	**0.013** DC > BC	0.10 (0.38)	0.14 (0.55)	0.927
	Median (IQR)	0 (0) ^a^	0 (1.00) ^A^		0 (0) ^a^	0 (0) ^B^	
***Daytime dysfunction***	Mean (SD)	1.54 (1.06)	1.44 (1.02)	0.453	1.37 (0.97)	1.40 (0.94)	0.761
	Median (IQR)	2.00 (1.00) ^a^	1.00 (1.00) ^A^		1.00 (1.00) ^a^	1.00 (1.00) ^A^	

the control group: before Covid; the case group: during Covid; SD = standard deviation; IQR = interquartile range. Results of ^Mann-Whitney U test. Bold indicates p < 0.05 for before and during Covid comparisons. Different lower or upper-case letters indicate p < 0.05 for painful and non-painful TMD comparisons

Table 3 reflects the mean/median OHIP scores for the BC and DC groups with painful and non-painful TMDs. Though no significant differences in total and domain OHIIP scores were observed with painful TMDs, the DC group exhibited significantly greater psychological discomfort and disability scores than the BC group when non-painful TMDs were present (DC > BC). For the two periods, PF patients had significantly greater total-OHIP and all domain scores when compared to NP patients (PF > NP).

**Table 3:** Mean/median OHIP scores for the control and case groups with painful and non-painful TMDs.

		Painful TMDs			Non-painful TMDs		
Variables		Before Covid (BC)	During Covid (DC)	P-value	Before Covid (BC)	During Covid (DC)	P-value
**OHIP-TMDs**							
***Total-OHIP***	Mean (SD)	43.60 (19.20)	42.77 (18.64)	0.656^	25.18 (18.24)	28.49 (17.91)	0.125^
	Median (IQR)	43.00 (29.75) ^a^	40.00 (30.00) ^A^		21.00 (28.00) ^b^	28.50 (26.00) ^B^	
***Functional limitation***	Mean (SD)	5.49 (2.16)	5.24 (2.35)	0.539^	3.09 (2.41)	3.18 (2.38)	0.760^
	Median (IQR)	6.00 (3.00) ^a^	6.00 (3.00) ^A^		3.00 (4.00) ^b^	3.00 (4.00) ^B^	
***Physical pain***	Mean (SD)	9.05 (4.32)	9.06 (4.20)	0.866^	4.37 (3.83)	4.54 (3.97)	0.809^
	Median (IQR)	9.00 (6.00) ^a^	9.00 (6.00) ^A^		3.50 (6.00) ^b^	4.00 (6.00) ^B^	
***Psychological discomfort***	Mean (SD)	9.82 (4.68)	9.84 (4.11)	0.795^	6.59 (4.57)	8.01 (4.60)	**0.022^** DC > BC
	Median (IQR)	11.00 (7.75) ^a^	10.00 (6.00) ^A^		6.00 (8.00) ^b^	8.00 (6.50) ^B^	
***Physical disability***	Mean (SD)	4.30 (2.11)	4.05 (2.13)	0.504^	2.34 (1.93)	2.57 (2.05)	0.429^
	Median (IQR)	4.00 (3.00) ^a^	4.00 (4.00) ^A^		2.00 (3.00) ^b^	2.00 (3.00) ^B^	
***Psychological disability***	Mean (SD)	8.87 (5.31)	8.33 (5.35)	0.440^	5.34 (5.03)	6.51 (4.76)	**0.034^** DC > BC
	Median (IQR)	8.00 (9.00) ^a^	8.00 (8.00) ^A^		4.00 (7.75) ^b^	6.00 (7.00) ^B^	
***Social disability***	Mean (SD)	2.29 (2.16)	2.47 (2.31)	0.641^	1.34 (1.87)	1.23 (1.64)	0.997^
	Median (IQR)	2.00 (4.00) ^a^	2.00 (4.00) ^A^		1.00 (2.00) ^b^	1.00 (2.00) ^B^	
***Handicap***	Mean (SD)	3.78 (2.44)	3.77 (2.47)	0.953^	2.09 (2.12)	2.45 (2.36)	0.297^
	Median (IQR)	4.0 (3.75) ^a^	4.00 (4.00) ^A^		2.00 (3.75) ^b^	2.00 (4.00) ^B^	

the control group: before Covid; the case group: during Covid; SD = standard deviation; IQR = interquartile range. Results of ^Mann-Whitney U test. Bold indicates p < 0.05 for before and during Covid comparisons. Different lower or upper-case letters indicate p < 0.05 for painful and non-painful TMD comparisons

Tables 4 and 5 reflect the outcomes of correlation and logistic regression analyses. For the BC and DC groups with painful/non-painful TMDs, total-DASS was found to be moderately correlated to total-PSQI and total-OHIP (*r*_*s*_ = 0.41–0.63). The relationship between total-PSQI and total-OHIP, albeit significant, was weak (*r*_*s*_ = 0.27–0.37) (Table 4). Both painful and non-painful TMDs were significantly associated with age, total-DASS, total-PSQI, and total-OHIP but not gender and the pandemic in the univariate model (Table 5). With the multivariate analysis, only age and total-OHIP predicted painful and non-painful TMDs. While ORs were > 1 for painful TMDs (age OR = 1.05; 95% CI = 1.03–1.07 and total-OHIP OR = 1.05; 95% CI = 1.04–1.07), they were < 1 for non-painful TMDs (age OR = 0.96; 95% CI = 0.94–0.97 and total-OHIP OR = 0.95; 95% CI = 0.94–0.96) (Table 5).

**Table 4:** Correlations between the different variables for the control (before Covid) and case (during Covid) groups

		Before Covid (BC)		During Covid (DC)	
TMD category	Variables	Total-DASS	Total-PSQI	Total-DASS	Total-PSQI
**Painful TMDs**	**Total-DASS**	-	-	-	-
	**Total-PSQI**	0.47**	-	0.56**	-
	**Total-OHIP**	0.61**	0.32**	0.54**	0.27**
**Non-painful TMDs**	**Total-DASS**	-	-	-	-
	**Total-PSQI**	0.47**	-	0.46**	-
	**Total-OHIP**	0.63**	0.37**	0.41**	0.32**

Results of Spearman’s correlation. *indicate p < 0.05, while ** indicates p < 0.01. Bold indicates moderate-to-strong correlations

**Table 5:** Univariate and multivariate logistic regression analyses for painful and non-painful TMDs.

	Univariate		Multivariate	
Variables	Odds ratio (95% CI)	P-value*	Odds ratio (95% CI)	P-value^
**Painful TMDs**				
**Pandemic**				
Before Covid	Reference	*-*	Reference	-
*During Covid*	0.84 (0.57–1.21)	0.345	-	-
**Gender**				
*Male*	Reference	**-**	Reference	**-**
*Female*	1.39 (0.86–2.25)	0.184	-	-
**Age**	1.05 (1.04–1.07)	**< 0.001**	1.05 (1.03–1.07)	**< 0.001**
**Total-DASS**	1.01 (1.00-1.02)	**0.002**	0.99 (0.98-1.00)	0.071
**Total-PSQI**	1.08 (1.02–1.14)	**0.006**	-	**-**
**Total-OHIP**	1.05 (1.04–1.06)	**< 0.001**	1.05 (1.04–1.07)	**< 0.001**
**Non-painful TMDs**				
**Pandemic**				
Before Covid	Reference	-	Reference	-
*During Covid*	1.20 (0.82–1.74)	0.345	-	-
**Gender**				
*Male*	Reference	-	Reference	-
*Female*	0.72 (0.45–1.17)	0.184	-	-
**Age**	0.95 (0.93–0.97)	**< 0.001**	0.96 (0.94–0.97)	**< 0.001**
**Total-DASS**	0.99 (0.98-1.00)	**0.002**	1.01 (1.00-1.02)	0.071
**Total-PSQI**	0.93 (0.88–0.98)	**0.006**	-	-
**Total-OHIP**	0.96 (0.94–0.97)	**< 0.001**	0.95 (0.94–0.96)	**< 0.001**

Results of univariate and multivariate logistic regression analysis. Bold indicates p < 0.05

## Discussion

The prevalence of painful TMDs among TMD patients and the psychological, sleep, and OHRQoL profiles of patients seeking TMD care before and during the Covid-19 pandemic were investigated. Additionally, the predictive factors for painful/non-painful TMDs including the Covid-19 pandemic were explored. As the prevalence of painful TMDs did not differ considerably before and during the pandemic, the first research hypothesis was not sustained. The second and third hypotheses were partly supported as significant variances in sleep and OHRQoL parameters were observed between the BC and DC groups with painful as well as non-painful TMDs, which were associated with age and total-OHIP. Only adult patients (≥ 18 years old) were selected for the study as the DC/TMD Axis I protocols are still being developed for children/adolescents [47]. Furthermore, most of the study measures had only been validated in adults. The OHIP for TMDs (OHIP-TMDs) was chosen over other OHIP instruments as generic OHRQoL measures have greater “floor effects” (no impact), lower sensitivity, specificity, and responsiveness than condition-specific ones [45]. Women constituted 80.5% and 82.0% of the BC and DC groups, corroborating the greater vulnerability of the female gender to TMDs [6, 7]. Nevertheless, no significant differences in age, gender, TMD subtypes, and categories were discerned between the BC and DC groups suggesting that TMD phenotype was not affected much by “impact events”, specifically the Covid-19 pandemic.

### Comparison between case and control patients

General distress, depression, anxiety, and stress levels did not vary substantially between the control (BC) and case (DC) groups, irrespective of the presence of TMD pain. The BC and DC groups with painful TMDs presented moderate depression (7–10 points), severe/extremely severe anxiety (8–10 + points), and moderate stress (10–12 points), whereas their counterparts with non-painful TMDs had mild depression (5–6 points), moderate anxiety (6–7 points), and mild stress (8–9 points) during both periods. Findings were consistent with prior studies concerning psychological distress among patients with differing TMD subtypes [16, 17]. Though psychological distress scores were comparable, the DC group had significantly greater sleep latency and use of sleep medication scores than the BC group when TMD pain was present. Therefore, patients with painful TMDs were taking longer to fall asleep and using more sleep medications during the pandemic which was consistent with the high prevalence of sleep problems during this period [48], The longer sleep duration and better sleep efficiency of the DC group could be contributed by the latter in addition to “lock-down” periods and “work-from-home” arrangements during the pandemic. Overall sleep quality was generally poor (total PSQI of ≥ 6 points) for patients with painful as well as non-painful TMDs and worsened slightly during the pandemic. Although no significant differences in total and domain OHIP scores were discerned between the BC and DC groups in the presence of TMD pain, psychological discomfort and disability domain scores of the DC group were significantly higher than the BC group with non-painful TMDs indicating greater intra-articular (TMJ) and function-related impairments in psychological well-being during the pandemic. Grievances include feeling worried, self-conscious, miserable, tense, upset, and depressed over jaw problems which could have been heightened by increased parafunctional activities during the pandemic [32]. While the findings of the present study differed from that of Lee et al. who reported elevated levels of psychological distress in TMD patients during the pandemic, it corroborated that of Mendonça et al. who determined that the Covid-19 pandemic did not worsen overall OHRQoL [30, 31]. The variance may be ascribed to dissimilarities in study design, sample size, TMD diagnostic criteria, assessment tools, race/ethnicity, socio-cultural as well as local measures adopted to contain the pandemic. Given their considerable heterogeneity, the studies could not be aptly compared.

### Comparison between patients with painful and non-painful TMDs

Among the three negative emotional states, only stress was constantly greater in patients with painful TMDs (PF) when contrasted to their counterparts with non-painful TMDs (NP) before and during the Covid-19 pandemic. While stress is the psychological and/or physical response to adverse events, depression and anxiety are the feelings of hopelessness/despair, and nervousness/apprehension correspondingly. Most TMD research had emphasized depression and anxiety, while stress as a construct is less frequently explored [16, 17, 49]. However, the three negative emotional states are interconnected, and chronic upregulation of the hypothalamic-pituitary-adrenal (HPA) stress axis as well as higher cortisol secretion had been associated with pain catastrophization, depression, and anxiety in TMD patients [50]. Contrary to other earlier studies, no significant differences in depression and anxiety were observed between PF and NP patients during the Covid-19 pandemic [16]. This phenomenon could be rationalized by the generally elevated levels of depression and anxiety during the pandemic [21, 22]. PF patients were also observed to have substantially poorer sleep (total-PSQI) and deficits in several sleep parameters when compared to NP patients during the pandemic period. This may be ascribed to the higher stress levels in PF patients, given the association between stress, insomnia, and sleep quality [8, 11, 51]. The significantly poorer OHRQoL of the PF patients both before and during the pandemic was consistent with the outcomes of prior studies [10, 11]. Painful TMDs were found to reduce physical and psychosocial functioning in both patient and community-based samples [10, 11, 52, 53].

### Correlation and regression analyses

Total-DASS was found to be moderately correlated to total-PSQI and total-OHIP independent of evaluation periods and the presence of TMD pain. Psychological distress thus affects both sleep quality as well as OHRQoL and necessitates assessment during TMD management. As psychological distress and psychological well-being are interconnected, positive psychological interventions, such as mindfulness-based stress reduction and cognitive behavioral therapy, could be useful for lowering TMD pain and related psychosocial impairments [54]. Multivariate analyses revealed that age and total-OHIP were associated with the presence of TMD pain and dysfunction. While greater age and poorer OHRQoL were related to the presence of painful TMDs, the opposite was true for non-painful TMDs. Findings were congruent with the higher occurrence of painful TMDs in older TMD patients and intra-articular conditions in younger ones, which are accompanied by poorer and better OHRQoL respectively [10, 55]. Even so, the effect of age was small, modifying the odds of the two TMD categories by only 5% after controlling for possible confounders.

### Study limitations

This retrospective case-control study had a few limitations. First, a certain degree of sampling bias could be present as the controls and cases selected may not be truly uniform. While this was allayed by the similarities in age, gender, and TMD distributions between the BC and DC groups, differences in other socio-demographic variables such as education might exist. Although a prospective longitudinal cohort study design can yield more discerning information, the citywide TTI strategy for controlling the Covid-19 pandemic made this impractical. Additionally, the prevalence of painful TMDs among patients before and during the pandemic could not be ascertained with a single cohort. Second, as the study measures were self-reported by the patients, other information biases can also be an issue. These include recall, social desirability, and confirmation partialities [56]. Third, the significant and moderately strong correlations between greater general distress and poorer sleep quality as well as OHRQoL in TMD patients do not imply causation. Other factors including pain chronicity might play intermediary roles and additional research is needed to clarify the multifaceted interactions between pain, distress, sleep, and quality of life [57]. Similarly, the significant associations of age and OHRQoL with painful/non-painful TMDs in the multivariate analyses also do not indicate a causal relationship. Instead, it reinforced the importance of age-related physical/experiential changes and “biopsychosocial” well-being in the holistic care of TMD patients [58].

## Conclusion

The prevalence of painful TMDs among TMD patients was not increased by “impact events”, specifically the Covid-19 pandemic. The Covid-19 pandemic did not appear to exacerbate psychological distress, which was already elevated in patients with painful TMDs. However, it affected sleep, increasing sleep latency and the use of sleep medications in patients with painful TMDs, and heightened unease over jaw dysfunction in those with non-painful TMDs. Overall sleep quality was generally poor for patients with painful as well as non-painful TMDs and worsened slightly during the pandemic. For the pandemic period, patients with painful TMDs had considerably greater stress, poorer sleep, and worse OHRQoL than their counterparts with non-painful TMDs. Psychological distress was found to be moderately correlated to both sleep quality and OHRQoL. It needs to be assessed and addressed together with age-related physical/experiential changes as part of comprehensive TMD management, especially in unsettling and stressful times.

## Data Availability

The datasets generated and analysed during the current study are available from the corresponding author on reasonable request.

## Abbreviations

BC: Before the Covid-19 pandemic
CIs: Confidence intervals
CT: Combined
DASS-21: Depression, Anxiety, Stress Scales
DC: During the Covid-19 pandemic
DC/TMD: Diagnostic Criteria for Temporomandibular Disorders
HPA: Hypothalamic-pituitary-adrenal
IT: Intra-articular
NP: Non-painful temporomandibular disorders
OHIP: Oral Health Impact Profile
OHRQoL: Oral health-related quality of life
ORs: Odds ratios
PF: Painful temporomandibular disorders
PT: Pain-related
SQ: Symptom Questionnaire
TMDs: Temporomandibular disorders
TMJ: Temporomandibular joint
TTI: Test-trace-isolate
